# Enhanced Activity and Sustained Release of Protocatechuic Acid, a Natural Antibacterial Agent, from Hybrid Nanoformulations with Zinc Oxide Nanoparticles

**DOI:** 10.3390/ijms22105287

**Published:** 2021-05-18

**Authors:** Khaled AbouAitah, Urszula Piotrowska, Jacek Wojnarowicz, Anna Swiderska-Sroda, Ahmed H. H. El-Desoky, Witold Lojkowski

**Affiliations:** 1 Laboratory of Nanostructures and Nanomedicine, Institute of High Pressure Physics, Polish Academy of Sciences, Sokolowska St. 29/37, 01-142 Warsaw, Poland; jacek.wojnarowicz@tlen.pl (J.W.); a.swiderska-sroda@labnano.pl (A.S.-S.); 2 Medicinal and Aromatic Plants Research Department, Pharmaceutical and Drug Industries Research Division, National Research Centre (NRC), 33 El-Behouth St., Dokki, Giza 12622, Egypt; 3 Department of Biomaterials Chemistry, Chair of Analytical Chemistry and Biomaterials, Faculty of Pharmacy, Medical University of Warsaw, 1 Banacha St., 02-097 Warsaw, Poland; upiotrowska@wum.edu.pl; 4 Pharmacognosy Department, Pharmaceutical Industries Research Division, National Research Centre, 33 El-Behouth St., Dokki, Giza 12622, Egypt; wasseif@yahoo.com

**Keywords:** ZnO nanoparticles, protocatechuic acid prodrug, natural agents, hybrid nanoformulation, delivery system, sustained release, antibacterial effect, *Staphylococcus aureus*

## Abstract

Hybrid nanostructures can be developed with inorganic nanoparticles (NPs) such as zinc oxide (ZnO) and natural antibacterials. ZnO NPs can also exert antibacterial effects, and we used them here to examine their dual action in combination with a natural antibacterial agent, protocatechuic acid (PCA). To produce hybrid nanoformulations, we functionalized ZnO NPs with four types of silane organic molecules and successfully linked them to PCA. Physicochemical assessment confirmed PCA content up to ~18% in hybrid nanoformulations, with a PCA entrapment efficiency of ~72%, indicating successful connection. We then investigated the in vitro release kinetics and antibacterial effects of the hybrid against *Staphylococcus aureus*. PCA release from hybrid nanoformulations varied with silane surface modification. Within 98 h, only 8% of the total encapsulated PCA was released, suggesting sustained long-term release. We used nanoformulation solutions collected at days 3, 5, and 7 by disc diffusion or log reduction to evaluate their antibacterial effect against *S. aureus*. The hybrid nanoformulation showed efficient antibacterial and bactericidal effects that also depended on the surface modification and at a lower minimum inhibition concentration compared with the separate components. A hybrid nanoformulation of the PCA prodrug and ZnO NPs offers effective sustained-release inhibition of *S. aureus* growth.

## 1. Introduction

As microorganisms exhibit an increasing and troubling resistance to drug therapies, including antibiotics, the search for new solutions has become urgent. The growing prevalence of resistant bacteria poses a global public health threat [1] as pathogens become adapted to the most frequently used antibiotics [2]. Novel strategies are needed, especially those that rely on alternative drug sources, including natural agents from plant-derived compounds [3]. These agents offer a cost-effective diversity of chemical structures that can serve as a basis for drug development.

Among the microbes best known for developing resistance is *Staphylococcus aureus* (*S. aureus*), gram-positive bacteria associated with high morbidity, mortality, and biofilm formation [4]. *S. aureus* is a common culprit in infections of the skin and soft tissue, urinary tract, and bloodstream and in infective endocarditis, osteomyelitis, pulmonary infections, gastroenteritis, and toxic shock syndrome [5,6]. Incidence of these infections continues to grow, affecting medical care costs because of prolonged hospitalization [7]. The antibiotic used in these infections depends on whether the pathogen is susceptible or resistant (i.e., methicillin-resistant *S. aureus*; MRSA). In such cases, first-line antibiotics include vancomycin or one of the cephalosporins [8,9,10], which carry a high risk of serious adverse effects (nephrotoxicity, hypotension, hypersensitivity reactions in case of vancomycin) and antibiotic resistance [10]. New and safe antimicrobial drugs are urgently needed.

Nanostructures of various forms and composition are attracting interest in this area because of the unique characteristics they offer. Various nanoparticles (NPs) have been investigational targets as nanoplatforms, including silver, zinc oxide (ZnO), or titanium oxide NPs [11,12,13,14]. The US Food and Drug Administration lists ZnO NPs as “generally recognized as safe” and approved it under the category of food additives. These NPs are of special interest as a nanoplatform for drug delivery, especially because of their own antibacterial potential [15] arising from release of ionized zinc (Zn^2+^). The mechanism underlying these antibacterial effects [16,17,18] may involve cell membrane disruption, followed by NP penetration and multiple intracellular effects, including destruction of the cell membrane and mediation of reactive oxygen species (ROS), which are involved in cellular toxicity and apoptosis [16,19,20]. To date, studies of these NPs have focused on their direct antibacterial potential, but interest has also turned to using ZnO NPs in combination with other known antibacterial agents in hybrid nanoformulations. Thus far, these NPs have been conjugated with ciprofloxacin for testing against bacteria with multi-drug resistance [21], bound to the glucan-binding protein to trigger ROS production [22], functionalized by association with gallic and caffeic acids [23,24], and loaded with amoxicillin for use against several different bacterial species [25].

Among the 200,000 structures isolated from natural products are phenolic compounds with broad pharmacological activities, including in vivo and in vitro antibacterial effects. One of these phenolic compounds is the prodrug protocatechuic acid (3,4-dihydroxybenzoic acid, or PCA), which is found in fruits, vegetables, grains, herbs, and spices [26,27]. Because this compound is nontoxic at an orally therapeutic dose of 100 mg/kg, it represents a promising natural prodrug [27]. Studies with many different bacterial species suggest that PCA has a broad antibacterial potential [28,29,30,31]. A few studies have assessed the use of different nanostructures for drug delivery with PCA, e.g., for cancers [32,33].

Here, we sought to leverage the established safety and antibacterial effects of PCA by combining it with modified ZnO NPs in a nanoformulation that offers a sustained-release effect. We also assessed whether surface modification and loading of PCA in these hybrid nanoformulations affect antibacterial efficiency of the hybrid compared with ZnO NPs alone. We are aware of no existing reports on the preparation of a PCA-functionalized ZnO NP hybrid to enhance antibacterial activity against *S. aureus*. To test the antibacterial potential of this construct rather than conduct traditional antibacterial tests, we used aqueous solutions with the aim of mimicking the environment where the drug would be released from these nanoformulations to exert its therapeutic action, if any. For this purpose, we first performed in vitro drug release experiments at predetermined times and then collected the resulting solutions to use for assessing antibacterial effects. Thus, the findings we obtained reflect the antibacterial effects of PCA release from a nanoformulation with ZnO NPs. The promise of this approach is that it could serve as a platform for targeted local delivery of these drugs to the infected tissue or organ, perhaps has an antibacterial coating, for focused therapeutic action.

## 2. Results and Discussion

**Scheme 1:**
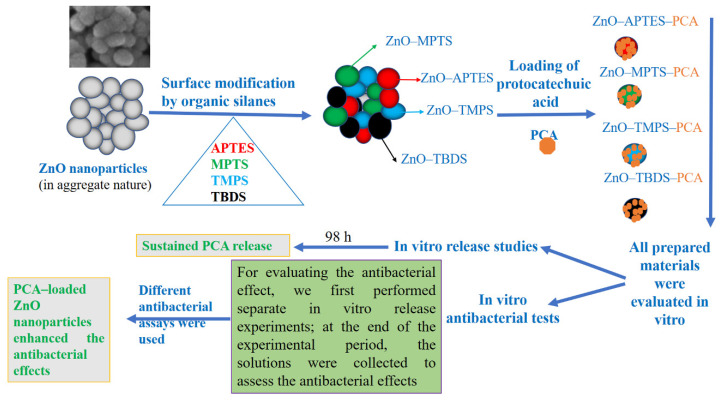
Schematic representation of all synthesis stages and biological evaluations against bacteria for a delivery system for the PCA prodrug depending on ZnO NPs.

Use of antibiotics as the main antibacterial agents against infection leads to significant adverse effects and antibiotic resistance [10], inspiring a search for new, safe alternatives. Here, we used advanced nanotechnology to evaluate the antibacterial effects of a nanoformulation containing an antibacterial prodrug and NPs. For this purpose, we modified ZnO NPs with different organic functionalities using a post-synthesis route of silane groups reacted with ZnO and then loaded with PCA, a naturally occurring phenolic compound. The ZnO NPs, functionalized ZnO NPs, and hybrid nanoformulations were then characterized by their physicochemical properties, and the nanoformulations were investigated for PCA release and antibacterial activity against *S. aureus*. The work is schematically represented in Scheme 1.

### 2.1. Characterization of Materials

#### 2.1.1. Morphological Structure and Elemental Measurements

**Figure 1:**
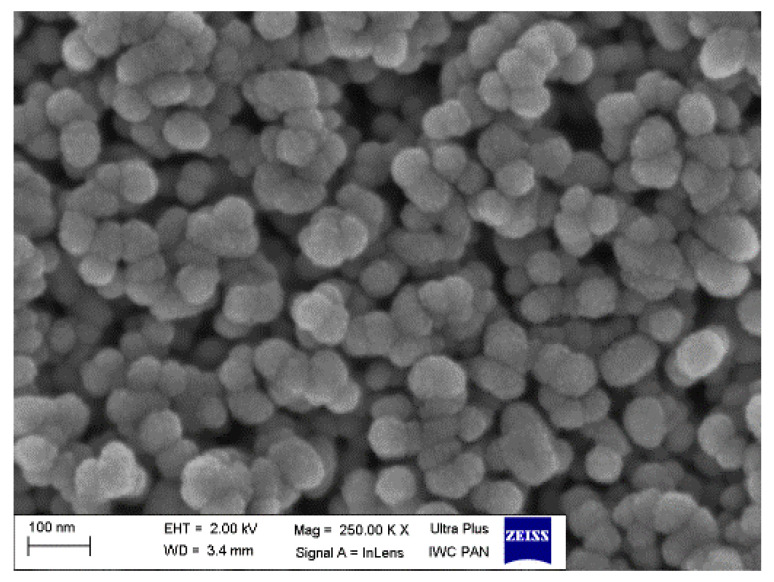
ZnO nanoparticles as visualized using FE-SEM.

The SEM image (Figure 1) shows that pure ZnO NPs are nearly spherical to oval in shape, with an aggregated nature. This aggregation seems to be formed by very small NPs of about 50 nm that combine to yield larger aggregates. This morphology is concordant with what was described using the original synthesis method of Wojnarowicz et al. [34].

We used EDX for the elemental analysis of materials at all preparation stages (Supplementary Materials Figure S1). As expected, the analysis showed that ZnO NPs consist of Zn and oxygen (O). Additionally, about 15 at. % of carbon (C) was detected. Earlier work indicated that some organic contribution from the alcohol used in solvothermal synthesis of a ZnO nano-powder may remain on the NP surface [35]. EDX analysis of surface-modified materials showed a carbon content at the level observed in pure ZnO NPs and below 1 at. % of silicon (Si). Moreover, the presence of other elements was detected as follows: nitrogen (N) in ZnO–APTES because of APTES amino groups; sulfur (S) in ZnO–MPTS; phosphorus (P) in ZnO–TMPS; and chlorine (Cl) in ZnO–TBDS. The presence of Si and other specific elements (N, S, P, and Cl) confirmed successful surface functionalization and represented proof of the presence of silane groups on or in ZnO NPs.

#### 2.1.2. Thermal Analysis of Materials

**Figure 2:**
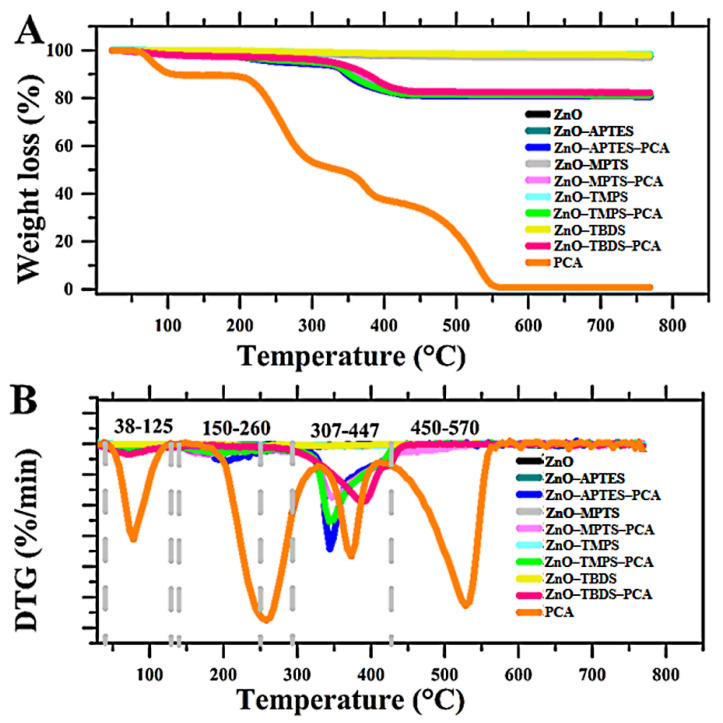
Thermal analysis results for materials at each stage by simultaneous thermal analysis, coupled with differential scanning calorimetry. (**A**) Weight loss measurements. (**B**) Derivative thermogravimetric (DTG) profiles. Materials were heated to 800 °C from RT at 10 °C/min in a mixture of artificial air and helium flowing through the furnace chamber.

Figure 2 and Table 1 show thermal analysis results for the materials at each stage. Pure ZnO NPs and all functionalized NPs showed similar thermal properties. One can see an almost simultaneous decrease in the mass of the material, falling below 3 wt.%. Derivative thermogravimetric (DTG) curves indicate intensification of mass change kinetics in the temperature range of 250–500 °C (Figure 2B) associated with the decomposition of organic substances in samples. As Table 1 shows, ZnO NPs and functionalized NPs did not differ significantly. Investigations of pure PCA showed that the prodrug decomposed during the experiment in 100% of the sample mass, with evidence of four process steps (Figure 2A,B). Mass loss was related to removing the water content (DTG peak centered at ~80 °C), and decomposition with DTG peaks centered at ~260 °C, 375 °C, and 530 °C. All hybrid nanoformulations demonstrated significant weight loss (about 20 wt.%) compared to ZnO NPs and functionalized ZnO NPs (less than 3 wt.%). Two stages of mass change were apparent during the heating to 800 °C (Figure 2A,B). The first appeared from RT up to 150 °C and was connected with water evaporation from the powder surface. The next significant mass loss was observed at 350–400 °C depending on the material type. After that, practically no mass change was observed up to the end of the experiment.

#### 2.1.3. DSC Characterization of Materials

**Figure 3:**
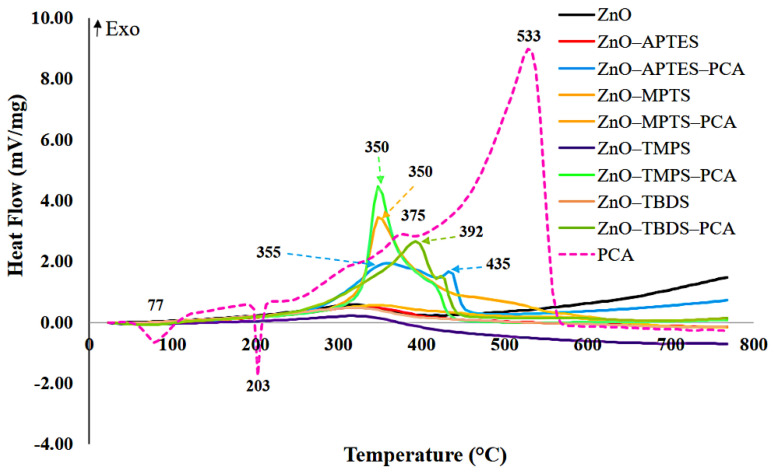
Differential scanning calorimetry analysis of ZnO NPs and surface-functionalized NPs. An exothermic process associated with mass loss is indicated.

DSC analysis of ZnO NPs and surface-functionalized NPs indicated an exothermic process during the experiments that was associated with mass loss (Figure 3). A broad exothermic peak centered at about 310 °C was detected in the case of ZnO NPs. After surface modification, the peaks were not symmetrical and shifted slightly up to a higher temperature compared to that for ZnO NPs. This difference most probably resulted from two overlapping processes: degradation of organic impurities in ZnO NPs and decomposition of silane components. For pure PCA, two endothermic peaks were present: a broad peak with a maximum at about 77 °C related to water evaporation and a sharp peak at about 200 °C related to PCA melting. Two exothermic signals followed, one that was small and broad with the extremum at about 375 °C and a sharper intense signal with a maximum at about 533 °C. These features are related to prodrug decomposition. We note that the DCS curve correlated with the kinetics of mass change observed on the DTG pictogram.

The DCS curves for nanoformulations presented a very weak endothermic peak arising from water evaporation at the beginning of the heating, but no endothermic peak related to melting of the prodrug was recorded. This observation suggests that PCA molecules present in nanoformulations have mainly an amorphous structure. For nanoformulations (ZnO–APTES–PCA, ZnO–MPTS–PCA, ZnO–TBDS–PCA and ZnO–TMPS–PCA), we observed an intense exothermic signal rising from two overlapping peaks. The first extremum of these non-symmetric peaks was present at 350–392 °C (depending on the sample) and reflected a decomposition process, as in the other materials. The second was seen at about 435 °C and was probably associated with the intense signal registered at 533 °C for pure PCA. The reduction in the process temperature may be traced to the amorphous state of PCA in nanoformulations. As can be seen, DSC changes for all nanoformulation materials were tracked with DTG data.

#### 2.1.4. XRD Characterization

**Figure 4:**
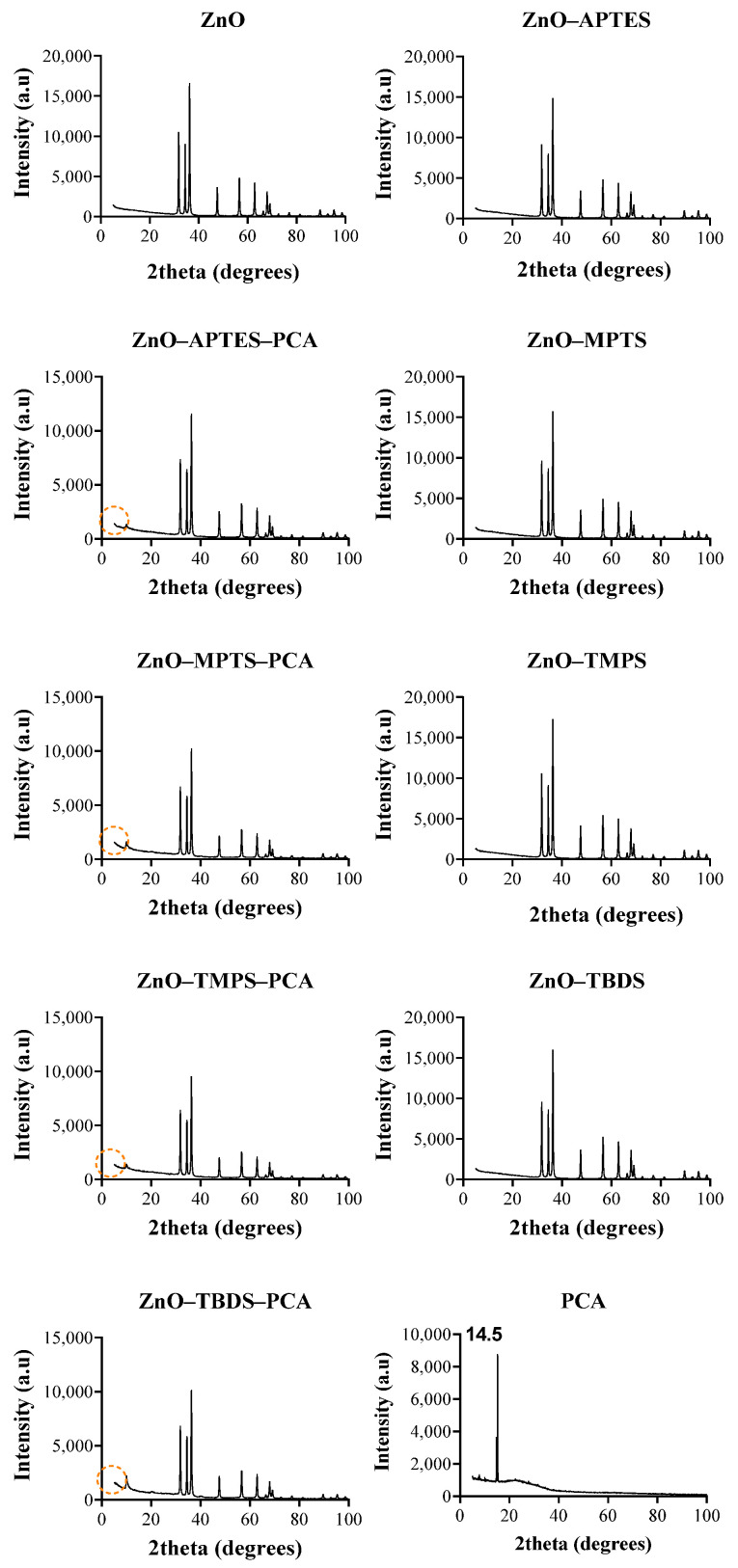
Results of X-ray diffraction analysis of dried powder materials for the PCA prodrug and before and after PCA loading in nanoformulations. The red-dotted circle indicates the presence of PCA in nanoformulations.

The XRD technique is a good method for characterizing and identifying the crystalline structural state of NPs and for distinguishing changes in materials at different preparation stages. As Figure 4 shows, we detected no foreign phases in any of the unfunctionalized or functionalized NPs or nanoformulations. The diffraction pattern of ZnO NPs was characterized by a hexagonal phase, which is in agreement with the original synthesis reported by Wojnarowicz et al. (see JCPDS card number 36-1451) [34,36]. The main peaks of the 2theta angle and the “hkl” obtained for ZnO NPs, functionalized ZnO NPs, and nanoformulations included the following: 31.7 Å (100), 34.4 Å (002), 36.2 Å (101), 47.5 Å (102), 56.5 Å (110), 63.0 Å (103), 66.3 Å (200), 68.0 Å (112), 69.0 Å (201), 72.6 Å (004), 77.0 Å (202), 81.4 Å (104), and 89.6 Å (203). No new peaks were detected for any of the functionalized NPs as a result of the organic functionalization process. Nanoformulations resulted in a new shift peak at 10.5 Å, corresponding to the main peak of the PCA pattern at 14.5 Å (Figure 4). Although this peak was seen with small intensity compared to the peak with free PCA, it indicates a successful loading process and that a small fraction of the prodrug could be attached to the NP surface. These results are in good agreement with the DSC results, both indicating that the PCA prodrug was loaded mainly in a non-crystalline state into functionalized ZnO NPs. However, some small amount of PCA was located on the NPs surface in the crystalline form, as indicated by the small diffraction peak.

#### 2.1.5. FTIR-ATR Analysis

**Figure 5:**
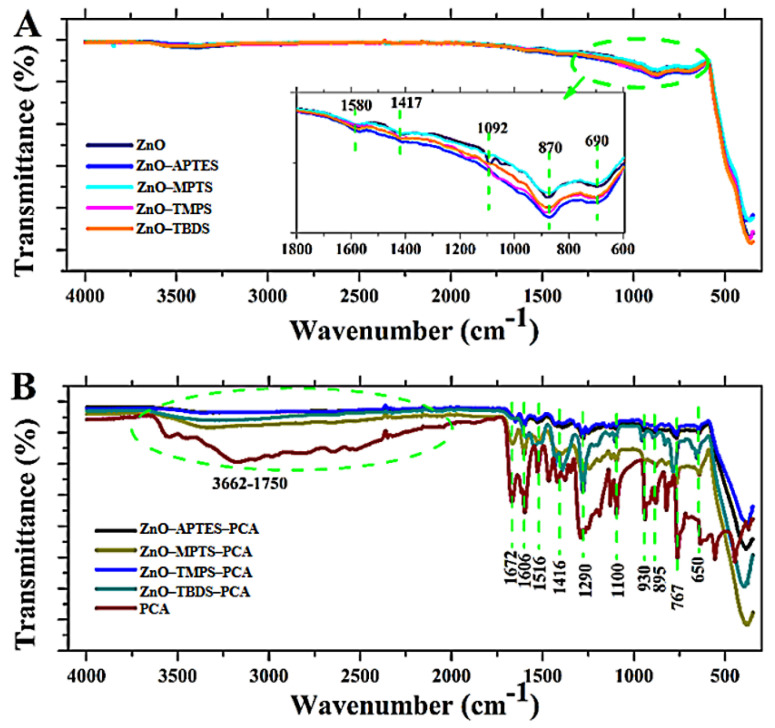
The spectra of ZnO NPs, modified ZnO NPs, and nanoformulations by Fourier-transform infrared spectroscopy. (**A**) ZnO NPs before and after surface modification. (**B**) Nanoformulations consisting of PCA and free PCA. In panel (**A**), the dotted green circle and lines show the changes following surface modifications with various silane groups compared to ZnO NPs. In panel (**B**), the dotted green circle and lines represent the detected new peaks in nanoformulations corresponding to free PCA.

Figure 5A presents the spectra obtained for ZnO NPs and functionalized ZnO NPs. The ZnO NP spectra indicated a broad band arising from -OH groups, exhibiting stretching vibrations at 3100–3600 cm^−1^. Bands that were detected at a maximum of 2900 and 2867 cm^−1^ were associated with C–H stretching vibrations, connected with post-processing impurities from ethylene glycol (C_2_H_4_(OH)_2_). Stretching vibrations from C=O groups produced absorption bands of 1570 and 1417 cm^−1^, whereas asymmetrical stretching vibrations from C–O groups produced bands of 1083–1030 cm^−1^. We found no significant change between ZnO NP spectra before and after functionalization. However, peaks with relative intensities at 870 and 690 cm^−1^ observed for functionalized materials might be attributable to Si–O bonds from silane functional groups, which usually appeared in this region [37]. Plausible explanations include the fact that the amount of silane in the samples was relatively small, as the thermogravimetry results indicated (Table 1), and that silane groups were presented inside NPs rather than on their surfaces, even below the limit of FTIR detection. FTIR spectra obtained for nanoformulations clearly gave proof of the presence of the prodrug (Figure 5B). The main bands detected for the pure prodrug (1672, 1606, 1516, 1416, 1290, 1100, 930, 895, 767, and 650 cm^−1^) were also seen in nanoformulations. We identified a main peak of 1672 cm^−1^ that we could assign to the stretching of C=C in PCA and a peak of 1290 cm^−1^ that we could assign to the carboxyl group (C=O) stretching [33,38]. In addition, peaks in the region between 3662–1750 cm^−1^ corresponding to free PCA could be attributed to O–H stretching vibrations and the PCA carboxyl group. This observation confirms that the main PCA molecules can be entrapped into nanoformulations (~18 wt.% by thermogravimetry results), with some fraction of the PCA prodrug still attached to the particle surface. These results are consonant with the findings using XRD.

#### 2.1.6. Zeta Potential Measurements of the Materials

**Figure 6:**
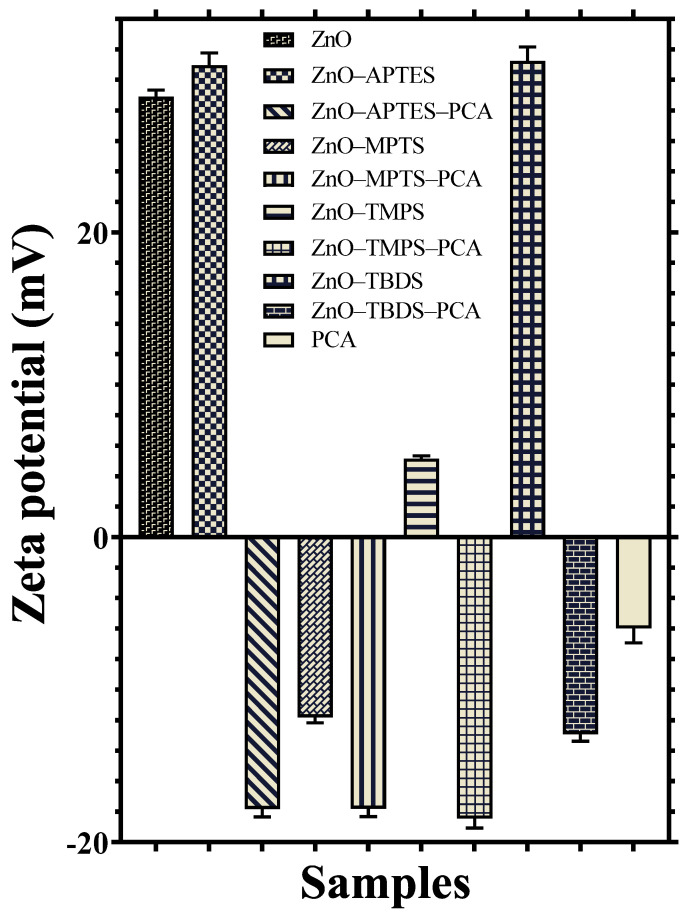
Zeta potential measurements in deionized water for all prepared materials before surface modification and after, along with PCA prodrug-loaded nanoformulations. The measurements were performed for all materials suspended in deionized water at pH 7.4.

As Figure 6 shows, surface modifications and PCA loading led to changes in surface charges carried by NPs suspended in deionized water. Generally, ZnO NPs and functionalized NPs did not differ. The zeta potential of ZnO NPs was positive (+28.8 mV), which is in agreement with previous studies [39,40,41]. Through further modification of the surface with APTES (ZnO–APTES) and TBDS (ZnO–TBDS), the functionalized ZnO–NPs had slightly higher positive charges of 30.8 ± 0.8 and 31.1 ± 0.9 mV, respectively. This contrasts with functionalization using MPTS (ZnO–MPTS), which had negative charges (−11.8 ± 0.35 mV). The NPs functionalized with TMPS (ZnO–TMPS) exhibited smaller positive charges of about 5.1 ± 0.2. All nanoformulations had a negative zeta potential ranging from −13 to −18 mV. The negative charges carried by nanoformulations were the result of the acidic nature of PCA, as we demonstrated with measurements of PCA alone, which showed a smaller negative zeta potential value (about −5.9 ± −0.9 mV). Overall, the results indicate that surface modification using various functional organic silanes and loading with PCA allowed for control of positive or negative charges on ZnO NPs in aqueous suspension. This finding is important because the ZnO NP surface affects other properties, such as cytotoxicity [40], by modulating Zn^2+^ ions release and antimicrobial effects [42].

### 2.2. Drug Loading

The loading and efficiency of PCA in nanoformulations was determined using the STA analysis. As Table 1 shows, the PCA loading content averaged between 16.8 to 17.8 wt.% depending on the surface functionalization of ZnO. In addition, nanoformulations did not differ significantly from each other. The efficiency for PCA loading in nanoformulations reached 71.3%, indicating successful preparation.

### 2.3. In Vitro Release and Kinetics

**Figure 7:**
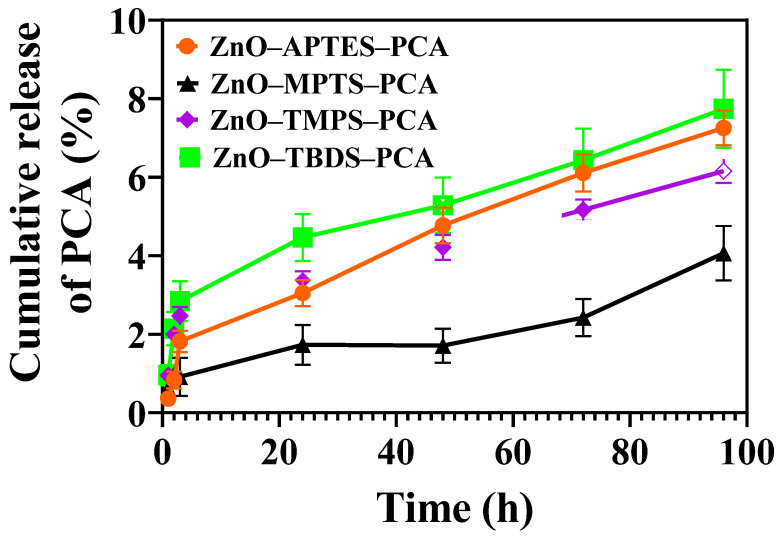
Release of the PCA prodrug from nanoformulations in the PBS medium (mean ± standard).

Release of PCA from hybrid nanoformulations can be modulated by organic functionality, further affecting antibacterial activity. The results suggest that surface functionalization influenced PCA release, as indicated by the release percentage shown in Figure 7 at the physiological pH of 7.4. The data demonstrated sustained release over time (almost 100 h), with varied release values. Release from nanoformulations was obtained, from highest to lowest, as follows: ZnO–TBDS–PCA > ZnO–APTES–PCA > ZnO–TMPS–PCA > ZnO–MPTS–PCA. However, the maximum percentage of PCA was only ~8% with the ZnO–TBDS–PCA nanoformulation within 98 h. These results suggest that PCA release can be extended for a long period because only less than 10% was released, a feature that is crucial when long-term antibacterial exposure is required. Barahuie et al. [32] demonstrated that the release of PCA from magnesium/aluminum-layered double hydroxide nanocomposite is pH-dependent, with a low release at pH 7.4 compared to pH values of 5.3 and 4.8. They also found that the release reached over 80% within 70 h. Similar results have been reported for PCA release from graphene oxide nanosheets, in which PCA release was pH-responsive [33]. The current published findings demonstrate a high release at pH 4.8 compared to 7.4, along with more than 30% released at pH 7.4 for 50 h [33]. Thus, the available data suggest that ZnO NPs of varying functionalization highly control PCA release. A plausible explanation is the strong interaction of PCA molecules with the functional surface groups distributed on modified ZnO NPs, making the release more controllable.

The results of PCA release kinetics from nanoformulations are shown in Table 2. The PCA release from the ZnO–APTES–PCA nanoformulation best fit the Baker–Lonsdale model; that of the ZnO–MPTS–PCA nanoformulation best fit the Hickson–Crowell model; and the ZnO–TMPS and ZnO–TBDS–PCA nanoformulations both fit best the Korsmeyer–Peppas model with a lag. In the comparison of the RE percentage (RE%), the values for RE% PCA were ordered as follows: 5.3% for ZnO–TBDS–PCA, 4.5% for ZnO–APTES–PCA, 4.2% for ZnO–TMPS–PCA, and 2.1% for ZnO–MPTS–PCA. The MDT reached 47.1 h for ZnO–MPTS–PCA, 35.6 h for ZnO–APTES–PCA, 30.2 h for ZnO–TBDS–PCA, and 30.2 h for ZnO–TMPS–PCA. The RR was found to be ~4.5%/h for ZnO–TBDS–PCA and ZnO–TMPS–PCA and ~2.2%/h for ZnO–APTES–PCA and ZnO–MPTS–PCA. These results suggest that PCA releases from nanoformulations through diffusion kinetics and that changing the surface chemistry of ZnO NPs using various organic functionalities can alter the RE, MDR, and RR for the PCA prodrug. Earlier findings indicated that PCA can be kinetically differed based on the nanocarrier nanoformulation used. In this context, Barahuie et al. [32] found that the pseudo-second-order model best described the kinetic release of PCA from the layered double hydroxide nanocomposite material. This model also applied to PCA release from loaded gadolinium and gold-layered double hydroxide nanocomposite and graphene [33,43].

### 2.4. Antibacterial Evaluations

#### 2.4.1. In Vitro Bacterial Activity by the Log Reduction Assay

**Figure 8:**
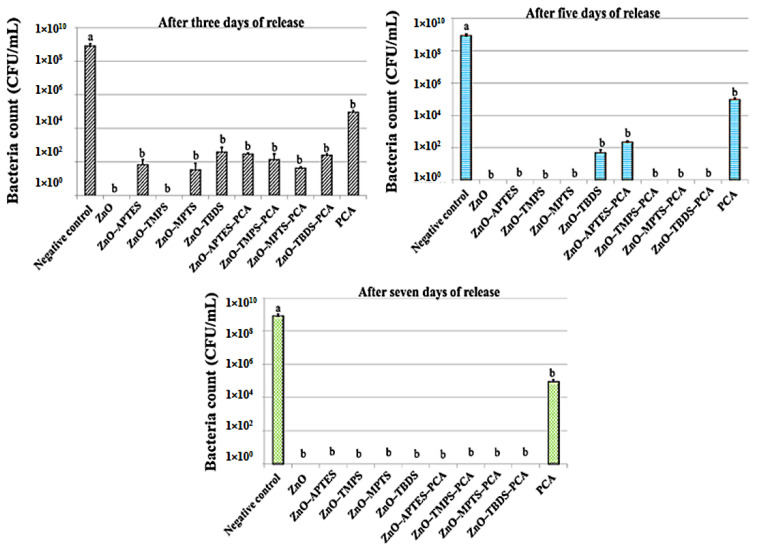
Results of the log reduction assay of ZnO NPs, surface-modified NPs, nanoformulations, PCA antimicrobial activity of the solutions (supernatants) obtained after three, five, and seven days against *S. aureus*. Columns labeled with different letters (a, b) had significantly different average values (*p* ≤ 0.05). Materials marked by the same letter (for instance, “a”) displayed no significant differences. Materials marked with different letters (for instance, “a” for one material and “b” for another material) displayed significant differences.

During the first stage of our investigation, we used the log reduction assay to evaluate ZnO NPs activity against *S. aureus* (Figure 8) and identified a significant antibacterial effect of NPs alone and of nanoformulations. The viable bacteria count decreased by log 6 to log 7 after three days of incubation compared to the negative control sample and by log 2 to log 3 compared to PCA alone. After five days of incubation, a bactericidal effect was seen for most of the samples. However, in the cases of functionalized particles of the ZnO–TBDS and ZnO–APTES–PCA nanoformulation, there was a slight bacterial growth along with the log 6 to log 7 reduction in viable bacteria counts. After seven days of release, no bacterial growth was noted for any of the tested materials. One possible explanation for that finding is Zn^2+^ ion release from ZnO NPs. Cierech et al. [44] used optical emission spectrometry with inductively coupled plasma to assess Zn^2+^ ion release from ZnO NPs (as used in our study). They found ion release even from a ZnO–PMMA nanocomposite at >4 mg/L. These results suggest that the significant antibacterial effect arises from the combination of released Zn^2+^ and PCA, leaving no statistically significant difference between ZnO, functionalized ZnO NPs, and nanoformulations. ZnO NPs are well-known for their unique antimicrobial activities in various microbial infections [16] and can act on bacteria by different mechanisms, including by triggering ROS damage to the cell and direct effects on the cell membrane [45,46].

#### 2.4.2. In Vitro Bacterial Activity by the Disc Diffusion Method

**Figure 9:**
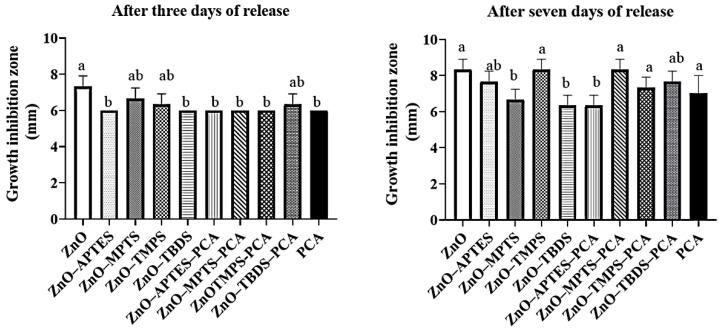
Antimicrobial activity of the solutions (supernatants) obtained after three and seven days of incubation of the tested materials at 37 °C in the PBS. Columns labeled with different letters (a, b) had significantly different average values (*p* ≤ 0.05). Materials marked by the same letter (for instance, “a”) displayed no significant differences. Materials marked with different letters (for instance, “a” for one material and “b” for another material) displayed significant differences.

We used the agar disc diffusion method to evaluate antimicrobial activity of the tested supernatants obtained from solutions after release in the PBS buffer. Figure 9 shows the resulting antibacterial activity for the tested NPs, nanoformulations, and PCA after three and seven days of incubation at 37 °C in the PBS. After three days of incubation, the most active were ZnO NPs (growth inhibition zone, 7.3 mm), probably because of rapid Zn (II) release. The ZnO–APTES, ZnO–TBDS, ZnO–APTES–PCA, ZnO–TMPS–PCA, and ZnO–MPTS–PCA samples did not differ from one another, but did differ significantly from ZnO NPs (*p* ≤ 0.05).

After seven days of release, all of the treatments exhibited antimicrobial activity. Antimicrobial activity did not differ significantly in comparisons of ZnO–APTES and ZnO–APTES–PCA, of ZnO–TMPS and ZnO–TMPS–PCA, of ZnO–MPTS–PCA and ZnO–TBDS and ZnO–TBDS–PCA. The observed antibacterial effect of ZnO NPs alone could be attributed to cell membrane disintegration or damage, genomic instability, and intracellular ROS production [15,17,23,47]. Moreover, we found statistically significant differences for the ZnO–MPTS (6.7 mm) and ZnO–MPTS–PCA nanoformulations (8.3 mm), with higher antimicrobial activity of the nanoformulation against *S. aureus* after seven days compared with ZnO NPs alone.

#### 2.4.3. In Vitro Bacterial Activity by MIC Determination

The tested solution’s supernatants of ZnO, functionalized ZnO NPs, PCA, and nanoformulations obtained after seven days of release were chosen for the biological tests because of their strong antimicrobial activities (Figure 8). The results are presented in Table 3. As the table shows, nanoformulations resulted in stronger antimicrobial activities vs. ZnO NPs (MIC, 2.07 mg/mL) or free PCA (MIC, 2.50 mg/mL). In case of ZnO–TMPS, ZnO–MPTS, and ZnO–TBDS, the effective initial concentration was 4.15 mg/mL. The reason that ZnO NPs had a greater effect than the modified NPs might be the presence of organic functionalization on the surface that delayed Zn^2+^ release from ZnO NPs. The expected effect of this would be decreased cytotoxicity [40], implying that surface modification can modulate cytotoxicity of ZnO NPs. Most of the nanoformulations decreased MIC to 1.03 mg/mL compared to other tested solutions. The phenolic structure of PCA covering the surface may not have allowed bacterial growth, and the Zn^2+^ ions from ZnO NPs may also have performed as expected. Lee et al. [23] assessed ZnO NPs functionalized with gallic acid, evaluating their antibacterial and antioxidant effects against MRSA. They found that gallic acid-loaded NPs enhanced antioxidant and antibacterial effects by reducing bacterial cell viability. Palanikumar et al. loaded amoxicillin into ZnO NPs and found enhanced antibacterial effects compared to ZnO NPs alone [25]. Furthermore, ciprofloxacin–conjugated ZnO NPs also are antibacterial against clinically isolated MRSA [21]. The results from our study indicate that a high affinity of the PCA phenolic structure underlies its antibacterial selectivity and activity, along with additional effects of Zn ions on the bacterial cell membrane.

## 3. Materials and Methods

### 3.1. Synthesis of ZnO NPs and Surface Modification

We synthesized ZnO NPs using a microwave-assisted solvothermal approach as previously described by Wojnarowicz et al. [34,35]. Four organic silanes were employed to functionalize the surface of ZnO NPs: 3-aminopropyltriethoxysilane (APTES; Sigma-Aldrich, St. Louis, MO, USA); a solution of 3-(trihydroxysilyl)propyl methylphosphonate monosodium salt (TMPS; Santa Cruz Biotechnology, Dallas, TX, USA); and (3-mercaptopropyl)trimethoxysilane (MPTS) and 98% tert-butyl(chloro) diphenyl-silane (TBDS; both from Arcos Organics, Geel, Belgium). To functionalize ZnO NPs, we added 1 g of ZnO NPs in toluene (100 mL) for APTES, MPTS, and TBDS, and in 18.2 MΩ ultrapure water (100 mL; Milli-Q^®^, Millipore, Darmstadt, Germany) for TMPS. The solutions were left under strong stirring for 10 min; then, 0.7 mL of APTES, MPTS, TMPS, or TBDS were added dropwise. After adjustment of the stirring speed to 300 rpm, the solution was maintained for 24 h at room temperature (RT). After this step, the solutions were filtered and washed with ultrapure water several times to remove unreacted molecules, followed by 24 h of drying at 60 °C. The resulting modified NPs were designated as ZnO¬–APTES, ZnO–MPTS, ZnO–TMPS, and ZnO–TBDS.

### 3.2. PCA Prodrug Loading

To prepare hybrid nanoformulations, PCA was loaded to functionalized ZnO NPs with a drug/carrier ratio of 1:3. For loading, we dissolved 100 mg PCA (Sigma-Aldrich) in dimethyl sulfoxide (Tedia, Fairfield, OH, USA) and ethanol (Fisher Scientific, Loughborough, UK) in a 1:1 ratio. A total of 300 mg of each functionalized ZnO NP was added to this solution, followed by 24 h of stirring at RT and then evaporation in a Rotavap oven at 60 °C (Büchi, Flawil, Switzerland). Next, we resuspended the resulting powder of PCA-loaded NPs in ultrapure water (20 mL), followed by evaporation for removal of any unloaded PCA, and repeated this process once more. The resulting product was dried in an oven for 12 h at 60 °C, and the hybrid nanoformulations yielded from this process were designated as ZnO–APTES–PCA, ZnO–MPTS–PCA, ZnO–TMPS–PCA, and ZnO–TBDS–PCA.

### 3.3. Characterization Techniques

To identify the resulting NPs at each stage, we used several techniques, including field emission (FE) scanning electron microscopy (SEM; Ultra Plus, Zeiss, Jena, Germany) coupled with energy dispersive X-ray spectroscopy (EDX) to characterize their morphology and perform elemental analysis. Samples used in SEM–EDX experiments were sputtered with gold. Powder X-ray diffraction (XRD; Almelo, Netherlands) using CuKα radiation (2θ range of 10,100°) was employed to identify the crystalline state. To identify surface functional groups, we applied Fourier-transform (FT) infrared (IR) spectroscopy using a Bruker Tensor 27 IR instrument (Bruker Corporation, Billerica, MA, USA) implemented with attenuated total reflectance using a Bruker Platinum ATR-Einheit A 255. To evaluate the thermal stability of the materials at each of the three preparation stages, we performed simultaneous thermal analysis (STA; 449 F1 Jupiter^®^, NETZSCH-Feinmahltechnik GmbH, Selb, Germany) using a combination of differential scanning calorimetry (DSC) and thermogravimetry. The samples were heated to 800 °C from RT at 10 °C/min in a mixture of artificial air and helium flowing through the furnace chamber. All experiments were performed under the same conditions. To measure the zeta potential of the particles in a deionized water suspension of each NP type, we used a Zetasizer Nano-ZS ZEN 3600 (Malvern Instruments Ltd., Malvern, UK).

### 3.4. In Vitro Release

Throughout the experiment, the powders from nanoformulations were placed with the phosphate-buffered saline (PBS) in glass bottles maintained at 37 °C in a water bath while being shaken at 150 rpm (shaker type 357, Elpin+, Lubawa, Poland). Up to 98 h, we would withdraw an aliquot from each bottle at predetermined timepoints and replace the withdrawn volume with fresh PBS buffer. After the aliquots were centrifuged to separate nanoformulation particles, the resulting supernatants were placed in new vials and stored at 4 °C until the UV–vis analysis. Absorbance was measured at 280 nm using the UV–vis technique (U-2900 spectrophotometer, Hitachi High-Technologies Corporation, Tokyo, Japan). Several measurements were performed for each sample, and the cumulative drug release of PCA from nanoformulations was calculated based on the following Equations (1) and (2):

% of mass released at time t = (mass (t)/total mass of the drug in the nanoformulation) × 100(1)

cumulative % = mass (t − 1) + mass (t) for the current measurement(2)

where mass (t) is the percentage release at the time of measurement and t − 1 is the percentage of the released drug at the previous timepoint.

For kinetic model fitting, we analyzed the data for release profiles using pharmacokinetics software (KinetDS3, developed at Jagiellonian University, Krakow, at the Faculty of Pharmacy’s Department of Pharmaceutical Technology and Biopharmaceutics). The kinetic parameters identified were release rate (RR), release efficiency (RE), and mean dissolution time (MDT) [48].

### 3.5. Antibacterial Evaluation Assays

#### 3.5.1. In Vitro Antimicrobial Assay by the Log Reduction Method

To evaluate the antimicrobial potential of NPs, we analyzed each component (functionalized ZnO NPs, nanoformulations, and PCA) using *Staphylococcus aureus* (*S. aureus*, ATCC 25923). Each ZnO NP, functionalized ZnO NP, and nanoformulation was immersed in the tryptic soy broth (TSB; Merck Millipore, Darmstadt, Germany) according to Kolmas et al. [49] to yield a final concentration of 8.3 mg/mL for ZnO NPs and modified ZnO NPs and 1.72 mg/mL for PCA (for nanoformulations). The samples were then incubated in a shaking water bath (Elpin+) at 100 rpm and 37 °C for three, five, or seven days. After incubation, the solutions in each glass bottle were transferred to a tube and centrifuged, and the supernatants were collected (with continually released PCA in the case of nanoformulations) to be used for antibacterial analysis. We inoculated the collected supernatants for the final bacterial concentration of ~2 × 10^3^ colony-forming units (CFU)/mL, followed by an 18-h incubation at 37 °C. Next, 100-μL samples withdrawn from the solutions were serially diluted and plated onto the TSB, followed by the second 18-h incubation at 37 °C. After analysis and counting of the resulting bacterial colonies, we prepared two control samples: one with bacterial cells (negative control, 2 × 10^3^ CFU/mL) and the second with pure PCA (1.72 mg/mL). All the experiments were conducted in triplicate.

#### 3.5.2. Antimicrobial Activities by the Disc Diffusion Method

The antimicrobial activities of ZnO NPs, functionalized ZnO NPs, PCA, and nanoformulations were assessed as described below using the disc diffusion method. Each of the test materials was dispersed in 10 mL PBS for a final concentration of 8.3 mg/mL for ZnO NPs and 1.72 mg/mL for PCA. The dispersions were maintained at 37 °C under shaking in a water bath at 100 rpm and then centrifuged at 3500 rpm for 5 min (MPW-54, MPW MED. Instruments, Warsaw, Poland). The supernatants drawn after this step were used in subsequent tests. For microbial suspensions of ~10^8^ CFU/mL, we used bacteria cultured overnight on solid media and inoculated onto agar plates with even spreading over the surface using a sterile swab. After 15 min of pre-incubation, 6-mm paper discs were impregnated with 20 µL of the supernatants and placed over the inoculated area, followed by an 18-h incubation at the temperature of 37 °C. After the incubation, we used calipers to measure the inhibition zone diameter on each plate. All the experiments were conducted in triplicate.

#### 3.5.3. Minimum Inhibitory Concentration (MIC) Determination

We used a standard microdilution method to determine the MIC for *S. aureus*. Initial bacterial inocula (5×10^5^ CFU/mL in the Mueller Hinton II Broth; Biomaxima, Lublin, Poland) were exposed in a 96-well plate to various dilutions of the supernatants (obtained after seven days of release of ZnO NPs, functionalized ZnO NPs, PCA, and hybrid nanoformulations into the PBS) and incubated at 37 °C for 18 h. The broth containing only bacterial cells was used as the negative control. All the experiments were conducted in triplicate.

### 3.6. Statistical Analysis

We performed analysis of variance (ANOVA) with post-hoc adjustment (Tukey) on the values obtained as averages of three replicates, with one-way ANOVA for drug loading parameters. Data quality was assessed using the Brown–Forsythe test, and we evaluated distribution normality with the Shapiro–Wilk test. A *p* ≤ 0.05 was set as the threshold for statistical significance, and all the analyses were conducted with Statistica (v. 13.1, StatSoft, Inc., Tulsa, OK, USA).

## 4. Conclusions

In this study, we produced hybrid antibacterial organic/inorganic nanoformulations by combining PCA and ZnO NPs functionalized by various organic silane groups. With this approach, we obtained a high PCA prodrug loading capacity of up to ~18 wt.%. In release tests, nanoformulations released only ~8% of the loaded PCA after 96 h, indicating a long-term release effect, with a rate that depended on the surface modification applied.

We evaluated the antibacterial and bactericidal effects of this nanoformulation against *S. aureus* using log reduction and disc diffusion assays. The antibacterial agent was tested using PBS solutions obtained after release, with separation of ZnO NPs and nanoformulations from the solution at predetermined time intervals of three, five, and seven days. A significant antibacterial and bactericidal effect was found for up to seven days of release, again, with the antibacterial effect depending on the surface modification of ZnO NPs. Results of the MIC assay gave clear evidence of improved antibacterial activity of hybrid nanoformulations against *S. aureus*, with hybrid nanoformulations exhibiting the lowest MIC values compared with ZnO NPs and functionalized ZnO NPs.

Overall, the hybrid organic/inorganic nanoformulation consisting of ZnO NPs loaded with PCA exhibited high long-term antibacterial and bactericidal effects against *S. aureus*. These findings suggest new possibilities for sustained antibacterial prodrug release in the treatment of bacterial infections and in antibacterial coatings beyond traditional approaches using ZnO NPs alone or free drugs.

## Figures and Tables

**Table 1:** Thermogravimetric analysis and PCA loading properties for surface-modified ZnO nanoparticles.

Material	Weight Loss (wt.%) ^a^	PCA Loading Content (wt.%) ^b^	Entrapment Efficiency ^c^
ZnO	2.55 ± 0.8 ^a^		
ZnO–APTES	2.02 ± 0.5 ^a^		
ZnO–APTES–PCA	19.6 ± 0.5 ^b^	17.6 ± 0.9 ^a^ as PCA	70.4 ± 3.7 ^a^
ZnO–MPTS	2.5 ± 0.4 ^a^		
ZnO–MPTS–PCA	19.8 ± 0.6 ^b^	17.2 ± 1.1 ^a^ as PCA	68.8 ± 4.4 ^a^
ZnO–TMPS	1.71 ± 0.0 ^a^		
ZnO–TMPS–PCA	19.5 ± 1.2 ^b^	17.8 ± 1.2 ^a^ as PCA	71.3 ± 5 ^a^
ZnO–TBDS	2.0 ± 0.4 ^a^		
ZnO–TBDS–PCA	18.8 ± 0.9 ^b^	16.8 ± 0.8 ^a^ as PCA	67.1 ± 3.3 ^a^

^a^ Weight calculated from thermogravimetry measurements. ^b,c^ PCA loading content and entrapment efficiency calculated based on weight loss obtained from thermogravimetry. EE (%) = PCA amount that theoretically calculated − amount of PCA that actually calculated from weight loss/theoretically calculated × 100. The superscript small letters indicate significant differences. The same letters indicate no significant differences, and different letters indicate a significance difference at *p* < 0.05. For example, there are no significant differences between ZnO and ZnO–APTES, and they are denoted as “a.” There are significant differences between ZnO and ZnOAPTES-PCA, and they marked by letters “a” and “b”, respectively.

**Table 2:** In vitro release criteria of PCA released from nanoformulations prepared based on ZnO NPs modified with various organic functionalities in the PBS.

Formulation	Release Characteristics	
ZnO–APTES–PCA	Best-fitting model	Baker–Lonsdale
	RE	4.5
	MDT	35.6
	RR	0.2
	R^2^	0.9901
ZnO–MPTS–PCA	Best-fitting model	Hickson–Crowell
	RE	2.1
	MDT	47.1
	RR	0.2
	R^2^	0.9236
ZnO–TMPS–PCA	Best-fitting model	Korsmeyer–Peppas with a lag
	RE	4.2
	MDT	30.2
	RR	0.4
	R^2^	0.9397
ZnO–TBDS–PCA	Best-fitting model	Korsmeyer-Peppas with a lag
	RE	5.3
	MDT	30.2
	RR	0.5
	R^2^	0.9484

RE, release efficiency, %; MDT, mean dissolution time, h; RR, release rate, %h.

**Table 3:** Biological activity of surface-modified ZnO NPs and nanoformulations consisting of the PCA prodrug.

**Material**	**Concentration of ZnO NPs in the Initial Solution, mg/mL**				
	**8.33**	**4.15**	**2.07**	**1.03**	**0.51**
	**Turbidity of the Culture Medium**				
ZnO	−	−	−	+	+
ZnO–APTES	−	−	+	+	+
ZnO–TMPS	−	−	+	+	+
ZnO–MPTS	−	−	+	+	+
ZnO–TBDS	−	−	+	+	+
**Material**	**Concentration of ZnO NPs in the Initial Solution, mg/mL**				
	**8.33**	**4.15**	**2.07**	**1.03**	**0.51**
	**Equal to the Concentration of PCA in the Initial Solution** **, mg/mL**				
	**1.72**	**0.86**	**0.43**	**0.21**	**0.10**
	**Turbidity of the Culture Medium**				
ZnO–APTES–PCA	−	−	−	+	+
ZnO–TMPS–PCA	−	−	−	−	+
ZnO–MPTS–PCA	−	−	−	−	+
ZnO–TBDS–PCA	−	−	−	−	+
Free PCA	MIC: 2.50 mg/mL				

+, turbid broth, indicates bacterial growth; −, clear medium, indicates no bacterial growth.

## Data Availability

Not applicable.

## Supplementary Materials

The following are available online at https://www.mdpi.com/article/10.3390/ijms22105287/s1, Figure S1: The elemental analysis by EDX of all the materials at different stages of preparation. In each material, we highlighted (in dotted-box) the element that should be seen in order to confirm the surface modification or PCA drug loading in the nanoformulations.
