# Supplementary material for: Enhanced Activity and Sustained Release of Protocatechuic Acid, a Natural Antibacterial Agent, from Hybrid Nanoformulations with Zinc Oxide Nanoparticles
Source: Int J Mol Sci. 2021 May 18;22(10):5287. doi: 10.3390/ijms22105287 (PMC8156785; doi:10.3390/ijms22105287)
Supplement: Supplementary file 1 [file ijms-22-05287-s001.zip › ijms-1182188-supplementary.pdf]

Supplementary Materials

Article

Enhanced activity and sustained release of protocatechuic acid, a natural antibacterial agent, from hybrid nanoformulations with zinc oxide nanoparticles

Khaled AbouAitah[1,2*], Urszula Piotrowska[3], Jacek Wojnarowicz[1], Anna Swiderska-Sroda[1], Ahmed H.H. El-Desoky[4] and Witold Lojkowski[1*]

1 Laboratory of Nanostructures and Nanomedicine, Institute of High Pressure Physics, Polish Academy of Sciences, Sokolowska 29/37, 01-142, Warsaw, Poland; e-mail: k.abouaitah@labnano.pl (K.A.); jacek.wojnarowicz@tlen.pl (J.W.); a.swiderska-sroda@labnano.pl (A.S.-S.); w.lojkowski@labnano.pl (W.L.).

2 Medicinal and Aromatic Plants Research Department, Pharmaceutical and Drug Industries Research Division, National Research Centre (NRC), P.C.12622, Dokki, Giza, Egypt; e-mail: k.abouaitah@nrc.sci.eg (K.A.)

3 Department of Biomaterials Chemistry, Chair of Analytical Chemistry and Biomaterials, Faculty of Pharmacy, Medical University of Warsaw, 1 Banacha St., 02-097, Warsaw, Poland, e-mail: upiotrowska@wum.edu.pl (U.P)

4 Pharmacognosy Department, Pharmaceutical Industries Research Division, National Research Centre, Dokki, P.O. 12622, Giza, Egypt

* Correspondence: k.abouaitah@labnano.pl (K.A.); w.lojkowski@labnano.pl (W.L.); Tel.: +48-22-888-0429 or +48-22-632-4302 (K.A. & W.L.); Fax: +48-22-632-4218(K.A. & W.L.)

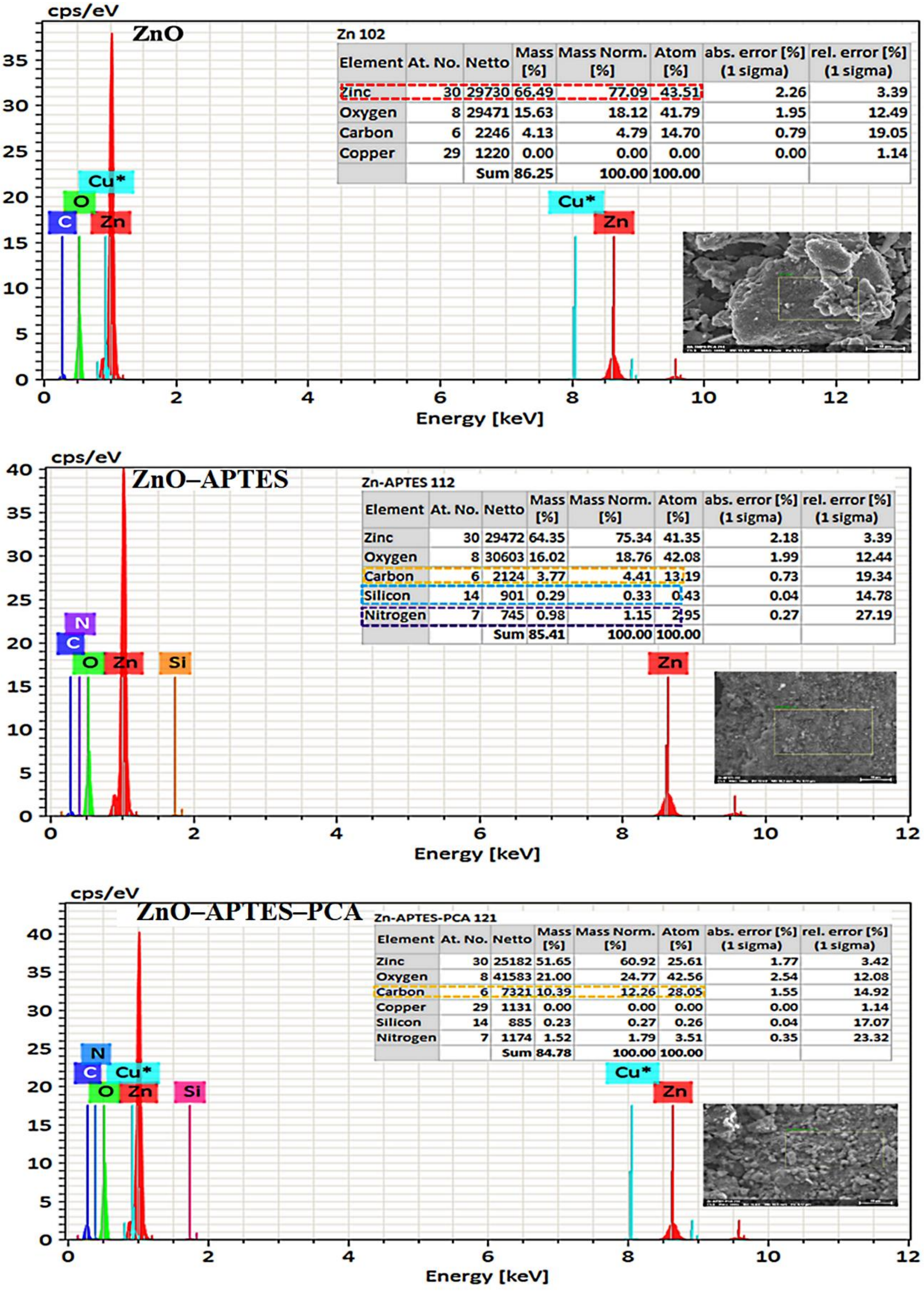

Zn 102

Element	At. No.	Netto	Mass [%]	Mass Norm. [%]	Atom [%]	abs. error [%] (1 sigma)	rel. error [%] (1 sigma)
Zinc	30	29730	66.49	77.09	43.51	2.26	3.39
Oxygen	8	29471	15.63	18.12	41.79	1.95	12.49
Carbon	6	2246	4.13	4.79	14.70	0.79	19.05
Copper	29	1220	0.00	0.00	0.00	0.00	1.14
		Sum	86.25	100.00	100.00		

Zn-APTES 112

Element	At. No.	Netto	Mass [%]	Mass Norm. [%]	Atom [%]	abs. error [%] (1 sigma)	rel. error [%] (1 sigma)
Zinc	30	29472	64.35	75.34	41.35	2.18	3.39
Oxygen	8	30603	16.02	18.76	42.08	1.99	12.44
Carbon	6	2124	3.77	4.41	13.19	0.73	19.34
Silicon	14	901	0.29	0.33	0.43	0.04	14.78
Nitrogen	7	745	0.98	1.15	2.95	0.27	27.19
		Sum	85.41	100.00	100.00		

Zn-APTES-PCA 121

Element	At. No.	Netto	Mass [%]	Mass Norm. [%]	Atom [%]	abs. error [%] (1 sigma)	rel. error [%] (1 sigma)
Zinc	30	25182	51.65	60.92	25.61	1.77	3.42
Oxygen	8	41583	21.00	24.77	42.56	2.54	12.08
Carbon	6	7321	10.39	12.26	28.05	1.55	14.92
Copper	29	1131	0.00	0.00	0.00	0.00	1.14
Silicon	14	885	0.23	0.27	0.26	0.04	17.07
Nitrogen	7	1174	1.52	1.79	3.51	0.35	23.32
		Sum	84.78	100.00	100.00		

Continued

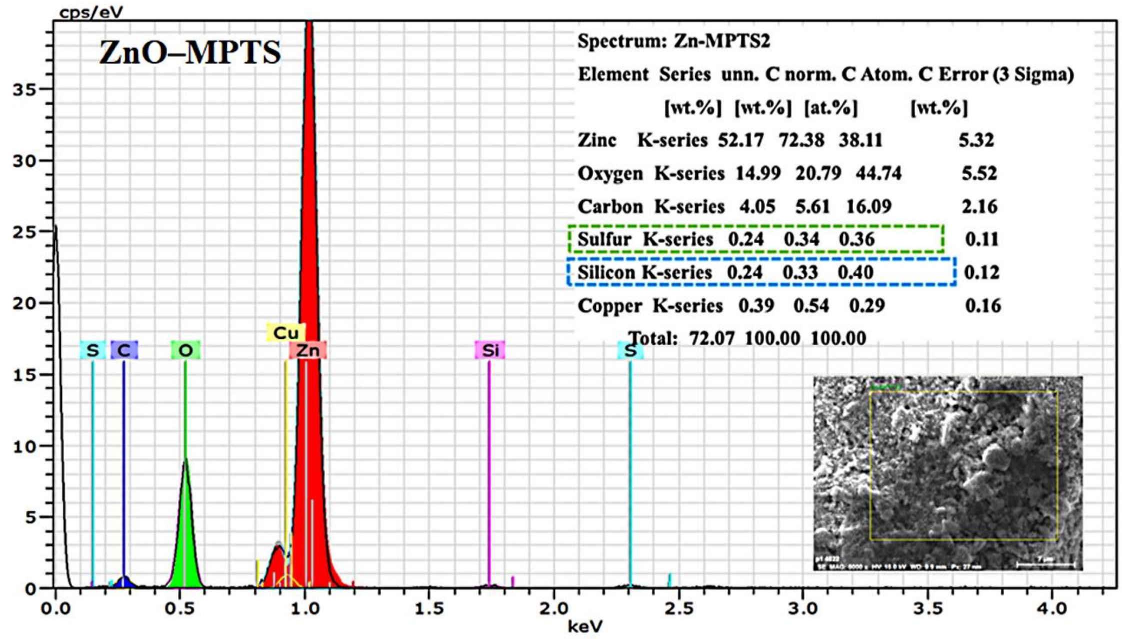
cps/eV
ZnO–MPTS
Spectrum: Zn-MPTS2
Element Series unn. C norm. C Atom. C Error (3 Sigma)
[wt.%] [wt.%] [at.%] [wt.%]
Zinc K-series 52.17 72.38 38.11 5.32
Oxygen K-series 14.99 20.79 44.74 5.52
Carbon K-series 4.05 5.61 16.09 2.16
Sulfur K-series 0.24 0.34 0.36 0.11
Silicon K-series 0.24 0.33 0.40 0.12
Copper K-series 0.39 0.54 0.29 0.16
Total: 72.07 100.00 100.00
S
C
O
Cu
Zn
Si
S
keV

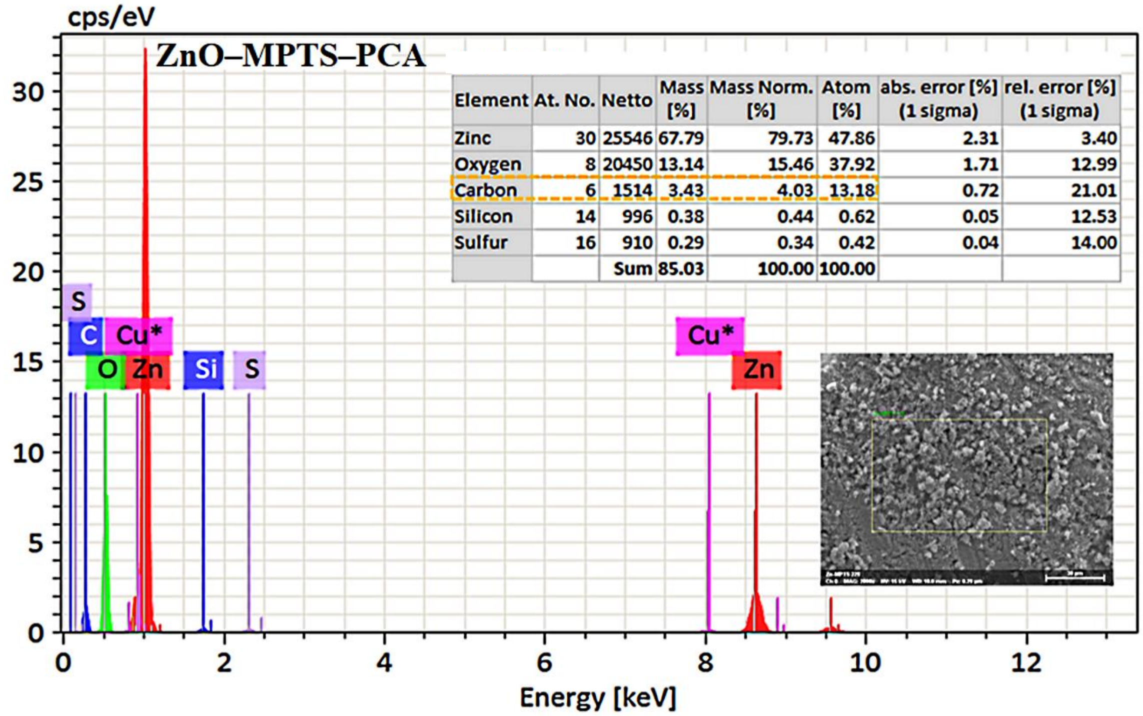
cps/eV
ZnO–MPTS–PCA
Element At. No. Netto Mass [%] Mass Norm. [%] Atom [%] abs. error [%] (1 sigma) rel. error [%] (1 sigma)
Zinc 30 25546 67.79 79.73 47.86 2.31 3.40
Oxygen 8 20450 13.14 15.46 37.92 1.71 12.99
Carbon 6 1514 3.43 4.03 13.18 0.72 21.01
Silicon 14 996 0.38 0.44 0.62 0.05 12.53
Sulfur 16 910 0.29 0.34 0.42 0.04 14.00
Sum 85.03 100.00 100.00
S
C
Cu*
O
Zn
Si
S
Cu*
Zn
Energy [keV]

Continued

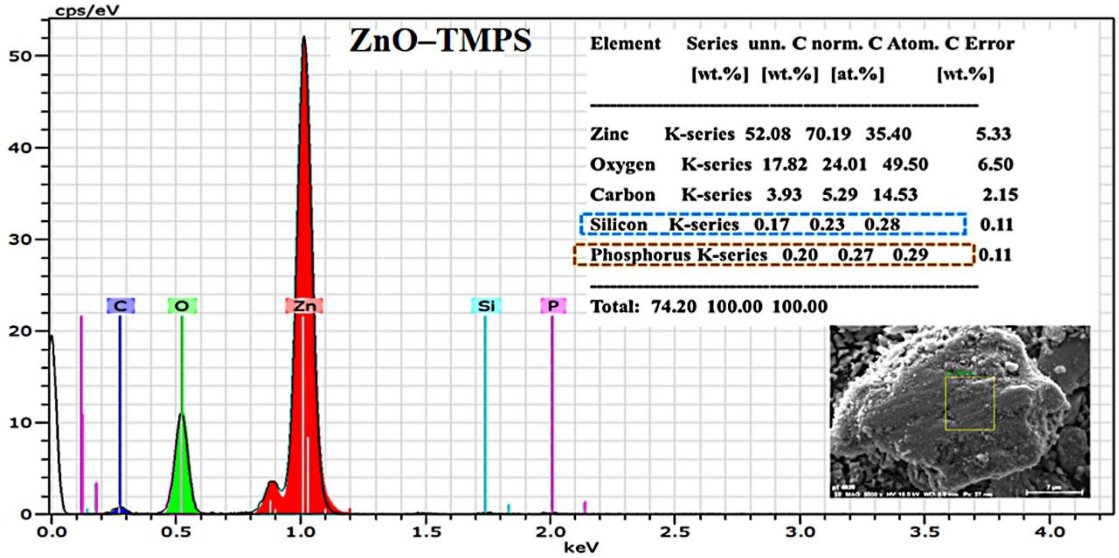
ZnO–TMPS
cps/eV
keV
C
O
Zn
Si
P
Element Series unn. C norm. C Atom. C Error
[wt.%] [wt.%] [at.%] [wt.%]
Zinc K-series 52.08 70.19 35.40 5.33
Oxygen K-series 17.82 24.01 49.50 6.50
Carbon K-series 3.93 5.29 14.53 2.15
Silicon K-series 0.17 0.23 0.28 0.11
Phosphorus K-series 0.20 0.27 0.29 0.11
Total: 74.20 100.00 100.00

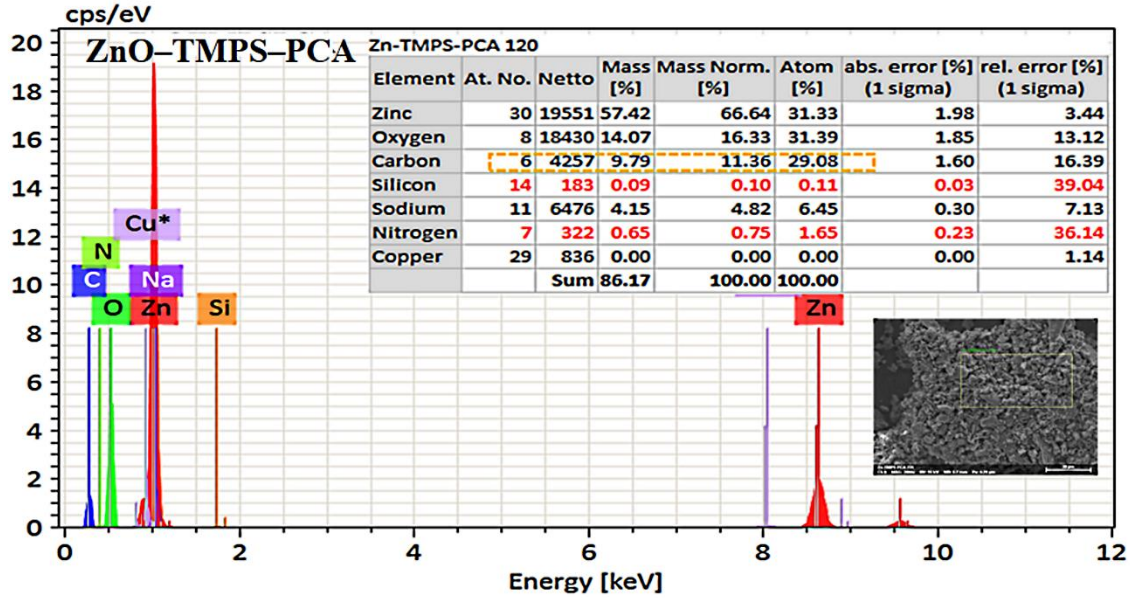
ZnO–TMPS–PCA
cps/eV
Energy [keV]
Cu*
N
C
Na
O
Zn
Si
Zn
Zn-TMPS-PCA 120
Element At. No. Netto Mass [%] Mass Norm. [%] Atom [%] abs. error [%] (1 sigma) rel. error [%] (1 sigma)
Zinc 30 19551 57.42 66.64 31.33 1.98 3.44
Oxygen 8 18430 14.07 16.33 31.39 1.85 13.12
Carbon 6 4257 9.79 11.36 29.08 1.60 16.39
Silicon 14 183 0.09 0.10 0.11 0.03 39.04
Sodium 11 6476 4.15 4.82 6.45 0.30 7.13
Nitrogen 7 322 0.65 0.75 1.65 0.23 36.14
Copper 29 836 0.00 0.00 0.00 0.00 1.14
Sum 86.17 100.00 100.00

Continued

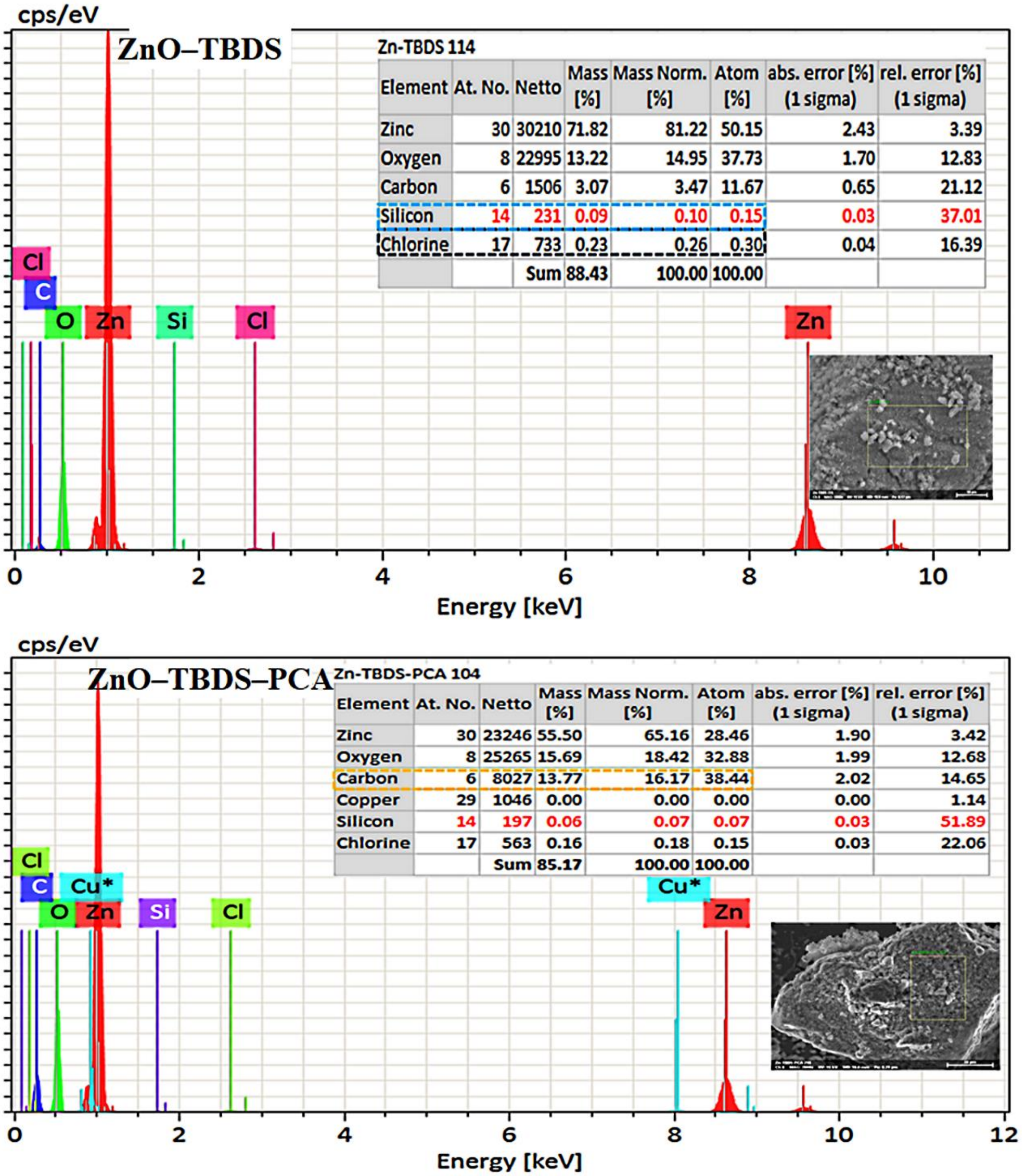

Zn-TBDS 114

Element	At. No.	Netto	Mass [%]	Mass Norm. [%]	Atom [%]	abs. error [%] (1 sigma)	rel. error [%] (1 sigma)
Zinc	30	30210	71.82	81.22	50.15	2.43	3.39
Oxygen	8	22995	13.22	14.95	37.73	1.70	12.83
Carbon	6	1506	3.07	3.47	11.67	0.65	21.12
Silicon	14	231	0.09	0.10	0.15	0.03	37.01
Chlorine	17	733	0.23	0.26	0.30	0.04	16.39
		Sum	88.43	100.00	100.00		

Zn-TBDS-PCA 104

Element	At. No.	Netto	Mass [%]	Mass Norm. [%]	Atom [%]	abs. error [%] (1 sigma)	rel. error [%] (1 sigma)
Zinc	30	23246	55.50	65.16	28.46	1.90	3.42
Oxygen	8	25265	15.69	18.42	32.88	1.99	12.68
Carbon	6	8027	13.77	16.17	38.44	2.02	14.65
Copper	29	1046	0.00	0.00	0.00	0.00	1.14
Silicon	14	197	0.06	0.07	0.07	0.03	51.89
Chlorine	17	563	0.16	0.18	0.15	0.03	22.06
		Sum	85.17	100.00	100.00		

Figure S1. The elemental analysis by EDX of all materials at different stages of preparations. In each material, we heighted (box) the element should be seen in order to confirm the surface modification or PCA drug loading in the nanoformulations.
